# WRINKLED1, a “Master Regulator” in Transcriptional Control of Plant Oil Biosynthesis

**DOI:** 10.3390/plants8070238

**Published:** 2019-07-22

**Authors:** Que Kong, Ling Yuan, Wei Ma

**Affiliations:** 1 School of Biological Sciences, Nanyang Technological University, Singapore 637551, Singapore; 2 Department of Plant and Soil Sciences, Kentucky Tobacco Research and Development Center, University of Kentucky, Lexington, KY 40546, USA

**Keywords:** Arabidopsis, transcription factor, plant oil biosynthesis, post-translational modifications, protein stability, protein-protein interaction

## Abstract

A majority of plant species generate and accumulate triacylglycerol (TAG) in their seeds, which is the main resource of carbon and energy supporting the process of seedling development. Plant seed oils have broad ranges of uses, being not only important for human diets but also renewable feedstock of industrial applications. The WRINKLED1 (WRI1) transcription factor is vital for the transcriptional control of plant oil biosynthetic pathways. Since the identification of the *Arabidopsis WRI1* gene (*AtWRI1*) fifteen years ago, tremendous progress has been made in understanding the functions of WRI1 at multiple levels, ranging from the identification of AtWRI1 target genes to location of the AtWRI1 binding motif, and from discovery of intrinsic structural disorder in WRI1 to fine-tuning of WRI1 modulation by post-translational modifications and protein-protein interactions. The expanding knowledge on the functional understanding of the WRI1 regulatory mechanism not only provides a clearer picture of transcriptional regulation of plant oil biosynthetic pathway, but also helps generate new strategies to better utilize WRI1 for developing novel oil crops.

## 1. Introduction

Plants mainly biosynthesize and accumulate plant oils (mostly as triacylglycerol, or TAG) in their seeds which serve as the carbon and energy resource for supporting seedling development. TAG, which is derived from seed oil and known to most people as vegetable oils, represents a highly energy-rich resource in nature (e.g., TAG has higher energy density compared to carbohydrates and proteins). In addition to the importance for the human diet, plant seed oils are also important as a renewable feedstock of industrial uses. Plant oils have broad applications for non-food industrial uses such as the manufacturing of soaps, detergents, and lubricants. Plant oils are also converted to biodiesel by chemical reactions. The global demand for vegetable oils is rapidly increasing and estimated to double by 2030 [1], escalating the necessity of increasing plant oil production to meet growing demand.

TAG biosynthesis involves cooperation between plastids and the endoplasmic reticulum (ER) [2]. Fatty acids biosynthesized in the plastids are exported for TAG assembly in the ER. The initial step of fatty acid biosynthesis starts with acetyl-CoA carboxylase, which converts acetyl-CoA and bicarbonate into malonyl-CoA. Fatty acids are then biosynthesized with two carbon augmentation via the fatty acid synthase (FAS) complex. Synthesized fatty acids are transported from plastids to ER in forms of acyl-CoA esters and TAG assembly in ER occurs mainly through the eukaryotic phospholipid biosynthetic pathway [3]. The final step of TAG biosynthesis, the conversion of diacylglycerol (DAG) to TAG, either using acyl-CoA or phospholipids, is mediated by diacylglycerol acyltransferase (DGAT) or phosphatidylcholine:diacylglycerol acyltransferase (PDAT) [4,5,6]. The regulatory mechanism of TAG biosynthesis is sophisticated and not understood thoroughly. Elucidation of the molecular mechanism of TAG biosynthesis is thus vital for both fundamental research of plant lipid biochemistry and design of new oil crops with the goal of increasing oil content. 

## 2. WRI1 Acts as a Key Transcriptional Regulator in Governing Plant Oil Biosynthesis

**Figure 1:**
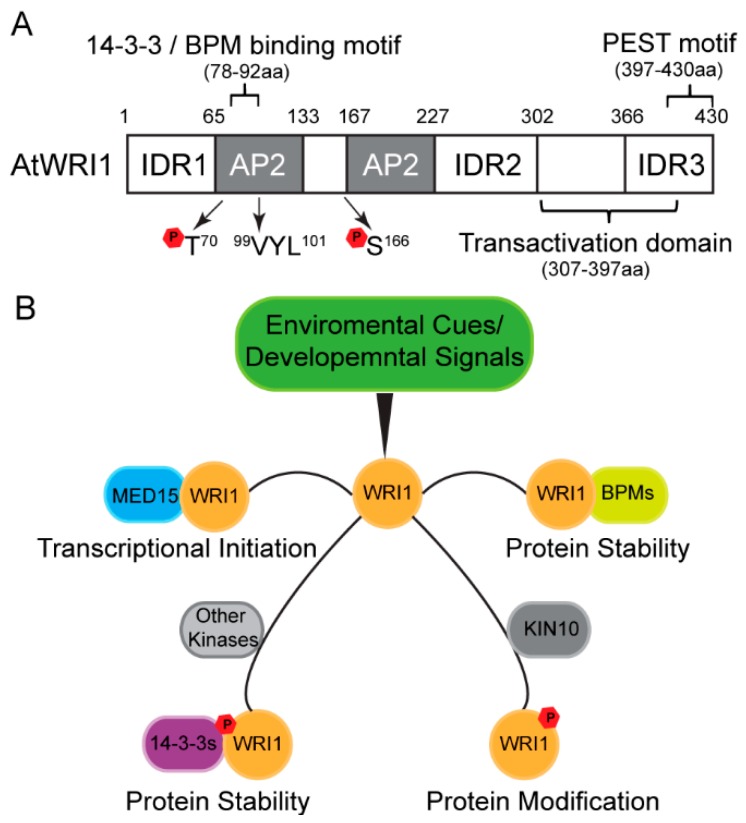
*Arabidopsis* WRINKLED1 (AtWRI1) structural features and regulatory mechanism of AtWRI1-mediated oil biosynthesis. (**A**) Schematic diagram of domains and motifs of AtWRI1, including two APETALA2 (AP2) domains, three intrinsically disordered regions (IDRs), a functional motif of “VYL”, the transactivation domain (TAD), the binding motifs of 14-3-3 and E3 ligase adaptor (BPM), PEST degradation signal sequence, and the KIN10 phosphorylation sites. (**B**) Post-translational regulatory mechanisms for AtWRI1 mediated by phosphorylation and newly identified interacting regulators, including Mediator Subunit 15 (MED15), BPMs, and kinase KIN10. The regulators modulate the protein stability and transcriptional activity of AtWRI1 via interactions and modifications, which in turn alters the gene expression of AtWRI1 targets.

A few transcriptional regulators, including WRINKLED1 (WRI1), are essential for plant oil biosynthesis. Pioneer work identifying *WRI1* was performed by the laboratory of Christoph Benning through Arabidopsis mutant screening. In 1998, Benning’s lab discovered *wri1-1*, the Arabidopsis loss-of-function mutant of *AtWRI1*, which displays a phenotype of 80% reduced seed oil content compared to the wild-type (WT) [7]. WRI1 is further characterized as a member of the APETALA2 (AP2) transcription factor family [8,9] (also see Figure 1A). In 2002, microarray analysis of developing seeds of WT and *wri1-1*, conducted in the laboratories of John Ohlrogge and Christoph Benning, indicated that the majority of the genes with decreased expression in the *wri1-1* mutant encode enzymes in the glycolytic and fatty acid biosynthetic pathways [10]. Subsequent work has validated numerous genes involved in glycolysis and fatty acid biosynthesis as direct targets of AtWRI1 [11,12] and characterized the AtWRI1 binding motif, the AW-box [12]. Given its importance, WRI1 is thus considered a “master regulator” for transcriptional regulation of TAG biosynthesis [1].

## 3. Regulators Involved in Mediating the Expression of *WRI1*

Central seed developmental regulators, LEAFY COTYLEDON1 (LEC1) and LEAFY COTYLEDON2 (LEC2), have been proposed to be key upstream transcription factors, which control the expression of *AtWRI1*. *AtWRI1* expression is elevated in *tnp* (a gain-of-function LEC1 mutant) [13] and *LEC1*-overexpression transgenic plants [14], implicating a potential function of LEC1 in activating *AtWRI1* expression [15,16]. The molecular and genetic evidence also suggests that LEC2 plays an important role in controlling the expression of *AtWRI1*. Reduced expression of *AtWRI1* was found in a *lec2* loss-of-function mutant [11]. Using inducible transgenic *LEC2* plants, Baud et al. have revealed that induction of *LEC2* activates *AtWRI1* expression [11]. The mechanism of LEC1/LEC2 controlling *WRI1* expression seems to be conserved in other plant species as well. For instance, the overexpression of soybean *LEC2* (*GmLEC2*) leads to the upregulated expression of soybean *WRI1* (*GmWRI1*) [17]. Expression of maize *WRI1* (*ZmWRI1*) is also elevated in transgenic maize embryos overexpressing maize *LEC1* (*ZmLEC1*). This mechanism is substantiated via co-expression of *ZmLEC1* with a *_promoter_ZmWRI1:GUS* reporter in maize cell culture. ZmLEC1 is able to activate *GUS* expression and elevate the GUS activity of a *_promoter_ZmWRI1:GUS* reporter in maize cell culture [18]. Nevertheless, the molecular basis of LEC1/LEC2 binding to the promoter of AtWRI1 has not been elucidated [15,16]. Pelletier et al. recently conducted chromatin immunoprecipitation (ChIP) followed by DNA microarray (ChIP-chip) assays and confirmed that *AtWRI1* is a direct target of LEC1 [19]. 

Molecular and genetic evidence also suggests that the *Arabidopsis* transcription factor FUSCA3 (FUS3) activates the expression of *AtWRI1*. Previous comparative microarray results indicated that the expression of *AtWRI1* is decreased in a *fus3* loss-of-function mutant compared to WT [20]. It has been hypothesized that FUS3 controls *AtWRI1* expression in a similar manner as LEC2, based on the functional redundancy between LEC2 and FUS3 [20]. Recently, Perry’s team conducted ChIP-chip assays and provided convincing evidence validating *AtWRI1* as a direct target gene of FUS3 [21]. The addition of sucrose activates the expression of *AtWRI1* in *Arabidopsis* seedlings [9]. AtWRI1 is also shown to trigger the expression of some sugar-responsive genes, suggesting a potential role of AtWRI1 in mediating the process of carbon flow to TAG [9]. LEC1 and LEC2 do not affect the expression of *FUS3*, hence the activation of *AtWRI1* (triggered by sucrose treatment) is concluded to be mediated by FUS3 directly [22]. 

Possible upstream WRI1 regulators, other than LEC1, LEC2, and FUS3, might exist in other plant species. For example, the expression of oil palm *LEC1* (*EgLEC1*), *EgLEC2,* and *EgFUS3* is low in the oil palm mesocarp where *EgWRI1* is highly expressed during fruit ripening, raising the speculation that expression of *EgWRI1* is regulated by novel regulators [23]. Recent work showed that EgNF-YA3, EgNF-YC2, and EgABI5 are able to bind to the *EgWRI1* promoter and activate *EgWRI1* expression [24]. 

The *Arabidopsis* transcription factor MYB89 has recently been identified as a new regulator, which represses *AtWRI1* expression. The overexpression of *MYB89* causes reduced expression of *AtWRI1*, and increased *AtWRI1* expression is detected in the *myb89* mutant. ChIP experiments further validated that MYB89 binds to the *AtWRI1* promoter, suggesting that *AtWRI1* is a direct target of MYB89 [25]. 

## 4. Molecular Regulatory Mechanism of AtWRI1 Activity

Since the identification of AtWRI1 in 2004 [8], advancements in understanding AtWRI1 function include identification of the AtWRI1 target genes in oil biosynthesis and characterization of the AtWRI1 binding motif. The last five years have seen significantly more publications on WRI1. However, little is known regarding other regulatory mechanisms mediating the activity of WRI1, such as post-translational modifications and interaction with other protein regulators. In addition, functional domains and motifs in WRI1 protein also remain to be characterized. 

The first WRI1-interacting partner has been identified through yeast two-hybrid (Y2H) screening. Using CULLIN3-based E3 ligase adaptor BTB/POZMATH 1 (BPM1) as prey led to the identification of AtWRI1 as a BPM1-interacting protein [26]. AtWRI1 interacts with other BPM proteins as well. The AtWRI1 protein is unstable and its assembly with E3 ligase adaptor BPMs mediates its degradation by the 26S proteasome [26]. This study bridges the connection between E3 ligase and plant oil biosynthesis through controlling WRI1 protein stability (Figure 1B).

Many proteins do not have a rigid three-dimensional structure, and intrinsic disordered regions (IDRs) have been broadly identified in eukaryotic proteins [27,28,29]. Recent work showed that AtWRI1 possesses three IDRs, as predicted by *in silico* analysis [30]. Functional characterization led to the identification of a PEST motif (a peptide signal for proteolysis), located in IDR3 of AtWRI1, and a transactivation domain (TAD) [30]. Both the IDR3-PEST motif and TAD are located at the C-terminus without overlapping (Figure 1A). Engineered AtWRI1s with either deletion of IDR3-PEST or mutations in possible phosphorylation sites in IDR3-PEST, results in increased protein stability and enhanced oil production compared to the native form of AtWRI1. Therefore, phosphorylation at the IDR3-PEST motif is proposed as a possible regulatory mechanism for AtWRI1 [30].

Multiple novel AtWRI1-interacting proteins that modulate AtWRI1 activity have recently been identified by several research groups. Ma et al. found that AtWRI1 physically interacts with 14-3-3 proteins in a Y2H and bimolecular fluorescence complementation (BiFC) assay *in planta* [31]. Overexpression of *14-3-3* increased AtWRI1-regulated oil biosynthesis and enhanced the protein stability and transcriptional activity of AtWRI1 (Figure 1B). Functional characterization revealed that the binding motifs of 14-3-3 and BPM overlap in AtWRI1 protein (Figure 1A). Hence, it is speculated that AtWRI1-14-3-3 interaction blocks BPM interaction with AtWRI1, or detachment of AtWRI1 from BPMs [31,32].

Zhai et al. found that KIN10 kinase physically interacts with AtWRI1 and triggers phosphorylation of AtWRI1 [33] (Figure 1B). AtWRI1 phosphorylation triggered by KIN10 is critical for the degradation of AtWRI1. The AtWRI1 mutant with mutations of two KIN10 phosphorylation residues (T70 and S166) abolishes KIN10-triggered phosphorylation and leads to enhanced protein stability of AtWRI1 [33] (Figure 1A). The proximity of the 14-3-3 binding motif and one KIN10 phosphorylation site in AtWRI1 suggests that these two modifications possibly overlap in the proteasome pathway [33]. Zhai et al. further found that trehalose 6-phosphate (T6P) plays a role in stabilizing the WRI1 protein and enhancing fatty acid biosynthesis by repressing KIN10 activity [34]. AtWRI1 protein level is higher in *adg1suc2* double mutant compared to WT, which is speculated via the repression KIN10-mediated phosphorylation on AtWRI1 [35]. 

The *Arabidopsis* mediator complex MED15 subunit is another novel candidate regulator, which physically interacts with AtWRI1 in multiple protein-protein interaction assays [36] (Figure 1B). *Arabidopsis* plants overexpressing *MED15* showed upregulation of AtWRI1 target genes in oil biosynthetic pathways. ChIP experiments subsequently demonstrated that MED15 binds to the promoter regions of these AtWRI1 target genes [36]. Nevertheless, transgenic *wri1* plants overexpressing *MED15* showed the unregulated expression of AtWRI1 target genes, suggesting that MED15 possibly interacts with other transcription factors to control the expression of AtWRI1 target genes [36].

## 5. *WRI1* Orthologs Identified in Various Plant Species

*WRI1* orthologs have been identified in a variety of plant species, both monocot and dicot, such as *Avena sativa* [37], *Brassica napus* [38], *Brachypodium distachyon* [39], *Camelina sativa* [40], *Cocos nucifera* [41], *Cyperus esculentus* [37], *Elaeis guineensis* [23,42], *Glycine max* [17,43], *Gossypium* spp [44], *Jatropha curcas* [45], *Persea americana* [46], *Ricinus communis* [47], *Solanum tuberosum* [37], and *Zea mays* [18,48]. Expression of *AtWRI1* and *WRI1* orthologs rescue the reduced-oil phenotypes of *wri1* loss-of-function mutants [8,40,42,45,48]. Many *WRI1* orthologs are highly expressed in developing seeds, similar to the expression pattern of *AtWRI1*. However, some *WRI1s* were found to be highly expressed in non-seed tissues. Expression of *EgWRI1* is enhanced in mesocarp (oil-producing tissues) and significantly increased during the fruit ripening process [23]. In addition, *PaWRI1* [46] and *CeWRI1* [37] display high expression in avocado mesocarp and nutsedge stem tuber, respectively. Protein structural features and functional motifs/domains, that have been characterized in AtWRI1 (e.g., “VYL” [42], IDR [30] and the PEST motif [30]), were found to be conserved in WRI1s identified from other plant species [30,39,40,42,45,49,50]. 

## 6. Newly Identified Targets of AtWRI1 That Are Not in Oil Biosynthetic Pathway

AtWRI1 is able to bind to the promoters of *PINs* (*PIN-FORMEDs*) at the AW-box, but binds the promoter of *GH3.3* (which encodes an enzyme participating in the auxin degradation) via a non-AW-box element, suggesting a role in modulating auxin homeostasis [51]. AtWRI1 is a homolog of CitAP2.10 (a *Citrus sinensis* AP2 transcription factor involved in (+)-valencene production) and able to activate the expression of *C. sinensis Terpene Synthase* 1 (*CsTPS1*) in a dual-luciferase assay [52]. At present, it is unknown how AtWRI1 regulation of these alternative target genes is associated with mediating plant development and growth. Nevertheless, the potential effects of alternative target genes of WRI1 on other plant physiological processes (e.g., auxin homeostasis [51]) needs to be taken into consideration in the application of WRI1 for bioengineering oil production. 

## 7. Applications of WRI1 in Bioengineering of Plant Oil Production

Transgenic plants overexpressing *AtWRI1* or *WRI1* orthologs have been shown to elevate seed oil content [8,18,38,39,41,45,53]. Overexpression of *AtWRI1* and *BdWRI1* elevate oil content in leaves of transgenic *Arabidopsis* and *Brachypodium* seedlings [39,54]. Using the embryo-preferred *OLEOSIN* (*OLE*) promoter to drive *ZmWRI1* expression led to significantly elevated seed oil content in transgenic maize, while no oil increase was detected when used the starch endosperm-specific *19 KD ZEIN* promoter to drive *ZmWRI1* expression [18]. In addition, selection of the *FUS3* promoter to drive *AtWRI1* expression in transgenic *Arabidopsis*, aiming to extend oil production during the mid-phase of seed development, is an effective way to enhance seed oil content [55].

Transient overproduction of AtWRI1 or WRI1 orthologs have also been successfully used for producing TAG in tobacco leaves [30,37,56]. The transient co-expression of *AtWRI1* and *DGAT1* in tobacco leaves has resulted in significantly increased oil content compared to the sole expression of *WRI1*, suggesting a synergistic effect between WRI1 and DGAT1 [56]. Transient ectopic expression of *WRI1* variants, including those with phosphorylation deficient mutations in the IDR3-PEST motif or removal of IDR3-PEST, lead to stabilized WRI1s and increased oil biosynthesis in tobacco leaves compared to overexpression of native WRI1 [30]. Transient co-expression of *AtWRI1* and 14-3-3 in tobacco leaves increases AtWRI1 stability and oil production [31]. The AtWRI1^K2RK3R^ mutant (with mutations in ubiquitination target sites) also displays increased protein stability and enhanced oil production compared to native AtWRI1 in a tobacco transient expression assay [33].

In addition, ectopic expression of some transcription factors from other plant species, which are capable of activating *WRI1* expression, have been shown to effectively increase oil content in seeds. For example, transgenic *Arabidopsis* plants overexpressing *GmZF351* or *GmDREBL* display enhanced seed oil contents. GmZF351 or GmDREBL have been found to be able to bind to the *AtWRI1* promoter [57,58]. Ectopic expression of *ZmLEC1* significantly enhances seed oil contents in *Arabidopsis*, *Camelina*, and maize [18,59].

Unusual fatty acids, e.g., hydroxy fatty acids (HFAs), have high value for industrial uses due to their special physical and chemical properties. However, transgenic plants overexpressing a hydroxylase gene accumulate a low amount of HFAs, while the seed oil content is decreased [60,61]. The reduction in seed oil was thought to be due to feedback inhibition of fatty acid synthesis [61]. To overcome this bottleneck, Adhikari et al. generated transgenic *Arabidopsis* plants co-expressing *WRI1* and a gene encoding a castor fatty acid hydroxylase, *RcFAH12*. The proportion of HFA and whole seed oil content in the transgenic plants significantly increased, suggesting WRI1 effectively rescues the feedback inhibition of fatty acid biosynthesis due to ectopic expression of a hydroxylase gene [62].

Irregular growth or cell death was observed in some transgenic plants overexpressing *WRI1* [8,15,39]. The selection of proper promoters to drive specific gene expression is, therefore, critical for bioengineering oil production in oil crops.

## 8. Future Perspectives

Since the identification of the *wri1-1* mutant in 1998 [7], the work in the past two decades confirms the important role of WRI1 in transcriptional control of plant fatty acid biosynthesis. However, there are still important questions to be addressed. For instance, the molecular mechanism regulating *WRI1* expression is still unclear. How seed development regulators, such as LEC1, LEC2, and FUS3, control *WRI1* expression also requires further elucidation. Alternative regulators may be involved in modulating *WRI1* expression in response to developmental or environmental signals. Transcription factors mediating the expression of *EgWRI1* were recently identified in oil palm [24]. Whether regulation of *WRI1* expression by these regulators is conserved in other plant species requires further investigation. Deeper investigations of the *WRI1* promoters from different plant species and the multi-layer gene regulatory network will address this question, as well as other questions related to the broader biological functions of WRI1.

Efforts have been initiated to connect the protein structural features with the molecular function of WRI1. Characterizations of the IDR, TAD, PEST motif, 14-3-3 and BPM binding motif, and KIN10 phosphorylation sites in AtWRI1 [30,31,33] are advancing our understanding of the WRI1 regulatory mechanism at the molecular level. In addition to more detailed characterization of the functional domains and motifs in the WRI1 protein, future work should focus on investigating WRI1 dynamics fine-tuned by protein-protein interactions and in response to cellular signals and environmental cues. Potential phosphorylation residues have been identified in AtWRI1 [30] and some have been experimentally shown to be vital for the function of AtWRI1 [30,33]. Continuing discovery and characterization of novel kinases involved in modifying AtWRI1 will be necessary. In addition, it has been speculated that phosphorylation might play dual roles in modulating the stability of AtWRI1 in response to different developmental signals during plant embryo development [31]. Thus, it will be interesting to see how modifications mediated by different upstream kinases and altered signals might be involved in the regulation of *AtWRI1*.

AtWRI1 has been recently found to bind the AW-box or new binding elements in promoters of targets not involved in glycolysis and fatty acid biosynthesis [51]. Recent work by Liu et al. found that AtWRI1 directly upregulates the expression of genes encoding *BIOTIN ATTACHMENT DOMAIN-CONTAINING* (*BADC*) proteins, which are responsible for inhibiting fatty acid biosynthesis [63]. This work might suggest a novel mechanism in WRI1 regulation of fatty acid synthesis. A deeper understanding of WRI1 on mediating the gene expression of the targets, which are not linked to oil production, might contribute to overcoming the undesirable effects associated with overexpressing *WRI1s* in transgenic plants [8,15,39]. Alternative approaches, such as protein engineering and genome editing, may be used to enhance WRI1 binding specificities to the target genes in oil biosynthetic pathways.
